# Evaluation of prognostic and predictive value of microtubule associated protein tau in two independent cohorts

**DOI:** 10.1186/bcr2937

**Published:** 2011-11-02

**Authors:** Maria T Baquero, Karen Lostritto, Mark D Gustavson, Kimberly A Bassi, Franck Appia, Robert L Camp, Annette M Molinaro, Lyndsay N Harris, David L Rimm

**Affiliations:** 1 Yale University School of Medicine, Department of Pathology, 310 Cedar St, New Haven, CT 06520, USA; 2 Yale University School of Medicine, Department of Computational Biology, 333 Cedar Street, New Haven, CT 06510, USA; 3 HistoRx, 35 Northeast Industrial Road, Branford, Connecticut, 06405, USA; 4 Sanofi-Aventis, International Clinical Trial Management/Oncology, 200 Crossing Blvd. Bridgewater, NJ 08807, USA; 5 Yale University School of Medicine, Department of Epidemiology and Public Health, 500 College St, New Haven, CT 06511, USA; 6 Yale University School of Medicine, Department of Clinical Oncology, 333 Cedar Street, New Haven, CT 06510, USA

**Keywords:** microtubule associated protein- tau (MAP-tau), metastatic breast cancer, taxanes, prognostic, predictive, quantitative analysis, immunohistochemistry

## Abstract

**Introduction:**

Microtubule associated proteins (MAPs) endogenously regulate microtubule stabilization and have been reported as prognostic and predictive markers for taxane response. The microtubule stabilizer, MAP-tau, has shown conflicting results. We quantitatively assessed MAP-tau expression in two independent breast cancer cohorts to determine prognostic and predictive value of this biomarker.

**Methods:**

MAP-tau expression was evaluated in the retrospective Yale University breast cancer cohort (n = 651) using tissue microarrays and also in the TAX 307 cohort, a clinical trial randomized for TAC versus FAC chemotherapy (n = 140), using conventional whole tissue sections. Expression was measured using the AQUA method for quantitative immunofluorescence. Scores were correlated with clinicopathologic variables, survival, and response to therapy.

**Results:**

Assessment of the Yale cohort using Cox univariate analysis indicated an improved overall survival (OS) in tumors with a positive correlation between high MAP-tau expression and overall survival (OS) (HR = 0.691, 95% CI = 0.489-0.974; *P *= 0.004). Kaplan Meier analysis showed 10-year survival for 65% of patients with high MAP-tau expression compared to 52% with low expression (*P *= .006). In TAX 307, high expression was associated with significantly longer median time to tumor progression (TTP) regardless of treatment arm (33.0 versus 23.4 months, *P *= 0.010) with mean TTP of 31.2 months. Response rates did not differ by MAP-tau expression (*P *= 0.518) or by treatment arm (*P *= 0.584).

**Conclusions:**

Quantitative measurement of MAP-tau expression has prognostic value in both cohorts, with high expression associated with longer TTP and OS. Differences by treatment arm or response rate in low versus high MAP-tau groups were not observed, indicating that MAP-tau is not associated with response to taxanes and is not a useful predictive marker for taxane-based chemotherapy.

## Introduction

Taxanes are microtubule stabilizing agents and potent cytotoxic compounds that have been recognized as highly effective chemotherapeutic agents [1,2]. However, varying degrees of benefit, with response rates ranging from 32% to 68% in the adjuvant and metastatic settings, suggest the critical need for a companion diagnostic to predict which patients are most likely to benefit from taxane therapy and which can be spared the cytotoxic effects of such therapy [3].

Taxanes induce mitotic arrest and tumor cell apoptosis through the hyper-stabilization of microtubules. Biomarkers that indicate the state of microtubule stability in the cell could be useful for predicting taxane response. Microtubule associated proteins (MAPs) are endogenous regulators of microtubule stability, functioning to promote or inhibit microtubule polymerization and determining subsequent cell cycle progression or mitotic arrest. These proteins may serve as potential candidates for a companion diagnostic.

MAP-tau (Tau) is a well characterized microtubule stabilizer that is responsible for the bundling, spacing, and assembly of microtubules [4-6]. MAP-tau may compete for taxane binding sites and/or may be involved in the cooperative binding of taxol to microtubules [7,8]. Initial reports evaluating MAP-tau as a predictive marker have been conflicting. Early studies measuring MAP-tau mRNA levels in the neoadjuvant setting found significantly lower levels in patients with pathologic complete response [7,9] but no correlation with pathologic complete response was observed in patients from a subset of the GEPARTRIO trial [10]. Similarly, in a subset of the Hellenic Cooperative Oncology Group (HeCOG) trial, MAP-tau mRNA expression status was found to be non-predictive of benefit from paclitaxel in the adjuvant setting [11]. When MAP-tau protein expression was evaluated, most often using traditional immunohistochemical methods, conflicting results were also found. Within the adjuvant setting, in the NSABP-B 28 randomized clinical trial, there was no prediction of benefit from paclitaxel but high MAP-tau expression was a positive prognostic marker for improved survival [12]. In advanced breast cancer patients, an early study of MAP-tau expression found no prediction of benefit from taxane therapy [13]. However, two additional studies of advanced breast cancer patients found high MAP-tau expression predictive for response to paclitaxel [14,15] and a positive prognostic marker for improved overall survival [15].

The goal of this study was to clarify the prognostic and predictive value of MAP-tau. Protein expression for MAP-tau was quantitatively assessed using two independent cohorts. Prognostic value was evaluated using a large Yale University retrospective cohort of untreated, primary breast cancer patients. Predictive value for MAP-tau was assessed using tumor tissue from TAX 307, a randomized clinical trial that examined patient response to the taxane, docetaxel, with docetaxel as the only variable. To date, no studies evaluating MAP-tau as a biomarker have assessed patient response using only taxane therapy as the randomized treatment variable. The TAX 307 trial randomized docetaxel-doxorubicin-cyclophosphamide (TAC) versus 5-fluorouracil-doxorubicin-cyclophosphamide (FAC) as first-line chemotherapy for metastatic breast cancer. Patients were allowed to receive prior adjuvant endocrine therapy (tamoxifen) and/or chemotherapy but no prior taxanes were allowed. In this trial, inclusion of docetaxel resulted in an improved response rate (*P *= 0.02) but did not improve time to tumor progression (TTP) (*P *= 0.51) or overall survival (*P *= 0.93) compared with FAC alone [16].

## Materials and methods

### Patient and cohort characteristics

Formalin-fixed paraffin-embedded primary breast cancer tumors resected from 651 patients at Yale University/New Haven hospital between 1962 and 1983 were obtained from the archives of the pathology department at Yale University (New Haven, CT, USA) and have been previously described in detail [17] (see Table S1 in Additional file 1). Specimens and associated clinical information were collected under informed consent under the ethics guidelines and approval of the Yale Human Investigation Committee under protocol #8219 to DLR.

The second cohort, a prospectively collected, randomized phase III clinical trial, compared TAC versus FAC [16]. Patients were enrolled between 1 January, 1998 and 31 December, 1999, with a total of 489 patients randomized to receive either FAC (75/50/500 mg/m^2^) or TAC (500/50/500 mg/m^2^) as first-line chemotherapy for metastatic breast cancer. Prior adjuvant chemotherapy (but not a taxane and not > 240 mg/m2 doxorubicin) was allowed. A total of 39% of patients received prior adjuvant chemotherapy of whom 11% had received anthracyclines previously. Patients may have also received prior adjuvant hormonal therapy (described in more detail in Results).

Cycles were repeated every three weeks for six to eight cycles, depending on cumulative dose of prior doxorubicin treatment. Median patient age was 54 years with median follow-up time of 30 months, median disease-free survival of 27 months, and median number of cycles of TAC or FAC equal to six.

Baseline characteristics were well balanced and major negative prognostic factors were similar in both arms. Specimens and associated clinical information were collected under informed consent under the ethics guidelines and approval of the Dana Farber Human Investigation Committee and Yale Human Investigation Committee under protocol # 0804003757 to LH. Tumor blocks were available for 140 patients from this trial and represented 28.6% of all clinical trial participants. The TAX 307 subgroup (TAX 307S) showed no differences in patient characteristics when FAC and TAC treatment randomization groups were compared (Table 1). In addition, no significant differences in clinical characteristics were observed between TAX 307S and the original TAX 307 cohort indicating TAX 307S to be a representative subset.

**Table 1:** Clinicopathologic characteristics of TAX 307S stratified by treatment (TAC vs.FAC)

Treatment group			
		
Variable	FAC^1 ^(n = 54)	TAC^2 ^(n = 54)	P*
**Menopausal status**			
Premenopausal	9 (16.7)	15 (27.8)	0.119
Postmenopausal	36 (66.7)	28 (51.9)	
Other	9 (16.7)	11 (20.4)	
**Tumor size (cm)**			
≤ 2	9 (16.7)	15 (27.8)	0.216
2-5	29 (53.7)	27 (50.0)	
≥ 5	12 (22.2)	10 (18.5)	
Other	4 (7.4)	2 (3.7)	
**Nodal status**			
Negative for node metastasis	16 (29.6)	17 (31.5)	0.874
Positive for node metastasis	29 (53.7)	33 (61.1)	
Other	9 (16.7)	4 (7.4)	
**Histology**			
Infiltrating ductal carcinoma	43 (79.6)	46 (85.2)	0.800
Infiltrating lobular carcinoma	5 (9.3)	5 (9.3)	
Other	6 (11.1)	3 (5.6)	
**Tumor grade**			
Well/moderately differentiated	18 (33.3)	13 (24.1)	0.148
Poorly/undifferentiated	27 (50.0)	32 (59.3)	
Other	4 (7.4)	1 (1.9)	
Unknown	5 (9.3)	8 (14.8)	
**ER status**			
ER negative	18 (33.3)	22 (40.7)	0.398
ER positive	27 (50.0)	23 (42.6)	
Other	9 (16.7)	9 (16.7)	
**PR status**			
PR negative	19 (35.2)	22 (40.7)	0.458
PR positive	25 (46.3)	21 (38.9)	
Other	10 (18.5)	11 (20.4)	
**Prior adjuvant chemotherapy**			
No therapy	29 (53.7)	26 (48.1)	0.565
Yes therapy	25 (46.3)	28 (51.9)	
**Prior adjuvant hormonal therapy**			
No therapy	31 (57.4)	40 (74.1)	0.070
Yes therapy	23 (42.6)	14 (25.9)	
**Prior metastatic hormonal therapy**			
No therapy	39 (72.2)	41 (75.9)	0.662
Yes therapy	15 (27.8)	13 (24.1)	
**Response to therapy**			
Complete response (CR)	3 (5.6)	5 (9.3)	0.5844
Partial response (PR)	25 (46.3)	23 (42.6)	
Stable disease (SD)	15 (27.8)	14 (25.9)	
Progressive disease (PD)	9 (16.7)	7 (13.0)	
Other	2 (3.7)	5 (9.3)	

*P is given for chi-square analysis. Statistically significant *P *values (*P *< 0.05) are in bold.

^1^FAC, 5-fluorouracil-doxorubicin-cyclophosphamide combination

^2^TAC, docetaxel-doxorubicin- cyclophosphamide combination

ER, estrogen receptor; PR, progesterone receptor.

### Tissue microarrays and whole tissue slides

Tissue microarrays (TMAs) were constructed as previously described [17]. In brief, tissue specimens were prepared for microarray format by selecting representative breast tumor areas from 651 formalin-fixed, paraffin-embedded primary tumor blocks using hematoxylin and eosin stained whole-section slides. Breast core samples 0.6 mm in diameter were arrayed in a recipient block. TMA internal controls consisted of normal breast tissue, liver tissue and formalin fixed, paraffin embedded cell lines A431, BAF3, BT474, BT549, HT29, MB 231, MB435, MB436, MB468, MCF7, SKBR3, SW480, and T47D. Culture conditions and cell-line TMA construction have been previously published in detail [18]. Additionally, a specialized Index Array was constructed to confirm assay reproducibility within both Yale University and TAX 307S cohorts and to normalize AQUA^® ^scores between different immunostaining run dates. Finally, a non-tumor TMA containing normal breast tissue was constructed from breast reduction mammoplasties using 110 unique patient samples with two-fold redundancy (n = 220). The TAX 307S cohort consisted of 140 conventional whole tissue (WT) slides of representative tumor tissue.

### Antibodies and immunofluorescence

Yale University cohort TMAs and TAX 307S WT slides were immunostained using MAP-tau monoclonal antibody, which recognizes all human MAP-tau isoforms (1:750; mouse monoclonal, clone 2B2.100/T1029; US Biological, Swampscott, MA, USA). This antibody has been validated by western blot analysis and siRNA knock down [7]. For TAX 307S, serial sections of the index array TMA were stained alongside both cohorts to confirm assay reproducibility. Normal breast epithelium in the Yale University cohort TMAs and the TAX 307S WT slides served as internal positive controls, while omission of the primary antibody served as the negative control for each immunostaining event. Quantitative immunofluorescence staining was performed as previously described in detail (See Additional File 2).

### TMA image capture and analysis

The AQUA method of quantitative immunofluorescence has been previously described [19]. The TMA cohorts were captured and analyzed using V1.6 of the AQUA^® ^software (HistoRx, Branford, CT, USA) on the PM2000 platform. Specific parameters related to the TMA data collection are found in Additional File 2.

### Whole tissue image capture and analysis

In contrast to TMA image acquisition and analysis, WT sections from the TAX 307 clinical trial cohort required a different approach for image capture and analysis. Based on the size and contours of each resection area per slide, an image acquisition matrix was created with fields of view (FOV) ranging from 4 to 486 discrete images per slide. To avoid sampling bias in tumor image selection and to address issues of potential MAP-tau tissue heterogeneity, all cytokeratin-stained regions (rather than a variable number of regions selected at random) were collected for each tissue and quantitatively analyzed. A total of 15,816 images were collected and assessed for MAP-tau expression from the 140 cases received from the TAX 307 clinical trial. Not all cases were available for evaluation with a total of 22 cases (15.7%) missing due to tissue loss during staining or incomplete clinical trial data.

### Statistical analysis

Average values for MAP-tau AQUA scores from the TMA were calculated from two-fold redundant samples and treated as independent continuous variables. The median expression level of MAP-tau from normal breast tissue served as the pre-defined cutpoint to differentiate high from low MAP-tau expression in both cohorts. Chi-square analysis was used to compare TAX 307S patient characteristics between FAC and TAC treatment groups to ensure intra-group comparability and to compare TAX 307S patient characteristics with those of the original TAX 307 clinical trial cohort. Survival curves for both cohorts were constructed using Kaplan Meier methods and the Cox-Mantel log-rank test was used to calculate the association between expression and survival. Two survival endpoints were used in this analysis. Overall survival (OS) was assessed for the Yale University cohort while TTP was evaluated for the TAX 307 clinical trial cohort. Cox proportional hazards regression analysis was used to determine which independent factors significantly impacted OS. Analyses used OS in the Yale University cohort and progression-free survival (PFS) in the TAX 307S cohort. To evaluate the association between patient response and MAP-tau expression levels, chi-square analysis was performed. Tau-by-treatment interaction was calculated to assess the relation between MAP-tau expression and docetaxel efficacy. All *P *values were based on two-sided testing and differences were considered significant at *P *< 0.05. Statistical analysis was performed using JMP Statistical Discovery Software, Version 7.0.1 (SAS Institute, Inc., Cary, NC, USA) and R, Version 2.8.0 (R Development Core Team).

## Results

### MAP-tau expression and distribution

In order to establish a cutpoint that could be used to differentiate high versus low MAP-tau expression in patients, MAP-tau was measured in normal epithelial ducts and lobules in TMA format (n = 220). Average MAP-tau expression scores in normal breast tissue showed mean and median AQUA scores of 489 and 462, respectively, with a score range of 157 to 1425. The median MAP-tau expression score in normal breast tissue was subsequently used in all analyses to differentiate high expressers (AQUA score ≥ 462) from low expressers (AQUA < 462). All subsequent AQUA scores were normalized to this AQUA score range using the index array TMA.

**Figure 1:**
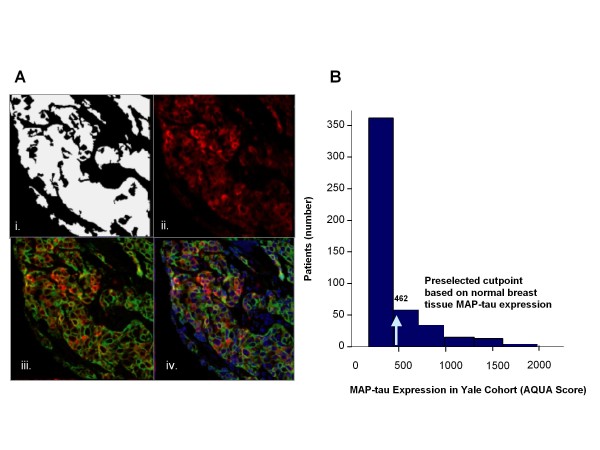
****MAP-tau expression and frequency distribution in Yale University cohort with two-fold redundancy**. (a) **Representative case from the Yale University cohort TMA showing cytoplasmic localization of MAP-tau within breast tumor tissue (two-fold redundancy; n = 651). (i) Pixel binary gating demarcating epithelial tumor area from stroma in order to define the tumor compartment; (ii) immunofluorescence of MAP-tau-Cy5 (red) expression pattern (continuous score of 789); (iii) target protein MAP-tau-Cy5 (red) colocalization with cytokeratin-Cy3, a marker for breast epithelia (green); (iv) Localization of nuclear DAPI (blue) relative to colocalization of target protein MAP-tau-Cy5 (red) with cytokeratin-Cy3, a marker for breast epithelia (green). Original magnification x20. **(b) **Yale frequency distribution of average MAP-tau expression scores in the Yale University cohort with preselected cutpoint 462, the median MAP-tau expression score observed in normal breast tissue and used to differentiate high expressors from low expressors.

We examined MAP-tau expression within the Yale University cohort (n = 651), and found cytoplasmic localization similar to our observations in normal breast tissue (Figure 1a). The frequency distribution of average MAP-tau expression scores in the Yale University cohort indicated mean and median AQUA scores of 498 and 210, respectively, with a range of 54 to 3017 (Figure 1b). A total of 480 cases had sufficient tumor tissue for analysis with 22% classified as high MAP-tau expressers compared with 78% as low expressers.

Estrogen receptor (ER)-negative cases showed significantly more frequent low MAP-tau (48.6%) compared with ER-positive cases (29.9%) (*P *< 0.0001) and this trend was mirrored for progesterone receptor (PR) status as well (Table 2). For HER2 expression, an inverse correlation between MAP-tau and human epidermal growth factor receptor (HER) 2-positive expression was observed with high MAP-tau present in only 2.4% of HER2-positive patients compared with 19.1% with high MAP-tau in HER2-negative cases. This was a particularly interesting observation as MAP-tau exists adjacent to HER2 on the 17q12 amplicon, yet rarely appears co-expressed in HER2-positive tumors. MAP-tau expression did not correlate with menopausal status, tumor size, nuclear grade, or nodal status (Table 2).

**Table 2:** Correlation between MAP-tau expression and clinicopathologic variables in the Yale University cohort

	MAP-tau expression		
		
Variable	Low (%) (AQUA score < 462)	High (%) (AQUA score > 462)	P*
**Menopausal status**			
Premenopausal	108 (22.8)	31 (6.5)	0.734
Postmenopausal	265 (55.9)	70 (14.8)	
			
**Tumor size**			
< 2 cm	128 (29.1)	31 (7.1)	0.535
2-5 cm	155 (35.2)	50 (11.4)	
> 5 cm	59 (13.4)	17 (3.9)	
			
**Nuclear grade**			
Small/uniform nuclei	60 (13.4)	15 (3.4)	0.225
Intermediate nuclei	182 (40.6)	61 (13.6)	
Large nuclei	107 (23.9)	23 (5.1)	
			
**Nodal status**			
Node positive	199 (41.8)	47 (9.9)	0.201
Node negative	175 (36.8)	55 (11.6)	
			
**ER**			
Negative	227 (48.6)	38 (8.1)	**< 0.0001**
Positive	140 (29.9)	62 (13.3)	
			
**PR**			
Negative	240 (52.4)	31 (6.8)	**< 0.0001**
Positive	120 (26.2)	67 (14.6)	
			
**HER2**			
Negative	283 (61.3)	88 (19.1)	**0.010**
Positive	80 (17.3)	11 (2.4)	

*P is given for chi-square analysis. Statistically significant *P *values (*P *< 0.05) are in bold.

ER, estrogen receptor; HER2, human epidermal growth factor receptor 2; PR, progesterone receptor.

### MAP-tau prognostic value in the Yale University cohort

**Figure 2:**
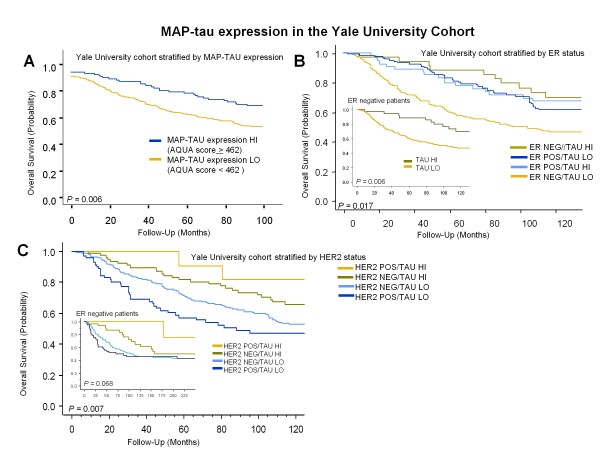
****Kaplan Meier survival analysis and MAP-tau expression in Yale University cohort**. (a) **Ten-year survival for high MAP-tau expression versus low expression for all invasive breast carcinoma patients in the Yale University cohort (n = 651). Survival rate for patients classified as high MAP-tau expressors (n = 94) was 68.3% compared with 52.9% for low expressors (n = 339; log-rank, *P *= 0.006). **(b) **Kaplan Meier survival for MAP-tau expression stratified by eatrogen receptor (ER) status in the Yale University cohort. For ER-negative patients (inset), high MAP-tau expression (n = 35) showed improved survival compared with low (n = 209) expression. **(c) **Ten-year survival for MAP-tau expression stratified by human epidermal growth factor receptor (HER) 2 status in the Yale University cohort. Patients classified as high MAP-Tau expressors showed improved survival compared with low expressors, regardless of HER2 status. For ER-negative patients stratified by HER2 status (inset), ER-negative patients with high MAP-tau expression (n = 30) trended toward improved survival, regardless of HER2 status, compared with low MAP-tau expressors (n = 56) (68.4% v 45.3%; log-rank, *P *= 0.068).

Patients with high MAP-tau expression (n = 94) showed improved survival compared with those with low expression (n = 339) (68.3% vs 52.9%, respectively; log-rank, *P *= 0.006, Figure 2a). When stratified by ER status, MAP-tau showed prognostic value in ER-negative but not in ER-positive patients (Figure 2b). In the ER-negative/high MAP-tau expressers (n = 35) we observed improved survival compared with low expressers (n = 209; 78% vs 42%; log-rank, *P *= 0.006). Similarly, patients stratified by HER2 status showed improved survival for high MAP-Tau/HER2 positive expression compared with low MAP-tau/HER2 positive expression (log rank *P *= 0.007, Figure 2c), although the coexpression of MAP-tau and HER2 was a rare event. Univariate analysis showed that high MAP-tau expression and ER and PR positive status were associated with significantly better OS (hazard ratio (HR) = 0.766 and 0.675; 95% confidence interval (CI), 0.598 to 0.981 and 0.524 to.871, *P *= 0.0005 and *P *< 0.0001, respectively), while large tumor size, nodal metastasis, increasing number of positive nodes, total nodes, and nuclear grade, were associated with worse OS (Table 3). In multivariate analysis, high MAP-tau expression was again associated with significantly improved OS. For patients with high MAP-tau expression, we observed a 24% reduction of risk (HR = 0.765; 95% CI, 0.598 to 0.957, *P *= 0.018; Table 4). In contrast, large tumor size, nodal metastasis, increasing total nodes, and positive HER2 status were associated with worse OS.

**Table 3:** Univariate analysis of tumor and clinical risk factors for overall survival in the Yale University cohort

	Yale University cohort			
		
Variable	No. of patients (%) (n = 651)	HR	95% CI	P*
**Age at diagnosis**	645 (99.1)	0.999	0.988-1.009	0.890
Unknown/missing	6 (0.9)			
				
**Menopausal status**				
Premenopausal	196 (30.1)	1.000		0.436
Postmenopausal	449 (69.0)	1.115	0.848-1.482	
Unknown/missing	6 (0.9)			
				
**Tumor size (cm)**				
≤ 2	215 (33.0)	1.000		**< 0.0001**
2-5	283 (43.5)	1.682	1.254-2.258	
≥ 5	101 (15.5)	2.911	2.074-4.086	
Other	52 (8.0)			
				
**Nodal status**				
Negative for node metastasis	327 (50.2)	1.000		**< 0.0001**
Positive for node metastasis	320 (49.2)	2.286	1.795-2.910	
Unknown/missing	4 (0.6)			
				
**Positive nodes**	320 (49.2)	1.022	1.001-1.042	**0.040**
				
**Total nodes**	625 (96.0)	0.978	0.961-0.994	**0.010**
Unknown/missing	26 (4.0)			
				
**Nuclear grade**				
Small/uniform nuclei	113 (17.4)	1.000		**0.0007**
Intermediate nuclei	315 (48.4)	1.231	0.854-1.818	
Large nuclei	170 (26.1)	1.594	1.192-2.123	
Other	53 (8.1)			
				
**ER status**				
ER negative	289 (44.4)	1.000		**0.0005**
ER positive	326 (50.1)	0.766	0.598-0.981	
Other	36 (5.5)			
				
**PR status**				
PR negative	294 (45.2)	1.000		**< 0.0001**
PR positive	302 (46.4)	0.675	0.524-0.871	
Other	55 (8.4)			
				
**HER2 status**				
HER2 negative	495 (76.0)	1.000		0.153
HER2 positive	109 (16.7)	1.270	1.048-1.317	
Other	47 (7.2)			
				
**MAP-tau expression**				
MAP-tau low expression	376 (57.8)	1.000		**0.0042**
MAP-tau high expression	104 (16.0)	0.691	0.489-0.974	
Unknown/missing	171 (26.3)			

* *P *is given for Cox univariate analysis, statistically significant *P *values (*P *< 0.05) are in bold, trending *P *values are in italics; CI, confidence interval; ER, estrogen receptor; HER2, human epidermal growth factor receptor 2; HR, hazard ratio; PR, progesterone receptor.

**Table 4:** Cox proportional hazards multivariate model for overall survival in the Yale University cohort

	Yale University cohort			
		
Variable	No. of patients (%) (n = 651)	HR	95% CI	P*
**Age at diagnosis**	645 (99.1)	1.004	0.981-1.026	0.718
Unknown	6 (0.9)			
				
**Menopausal status**				
Premenopausal	196 (30.1)	1.000		0.339
Postmenopausal	449 (69.0)	1.158	0.857-1.569	
Other	6 (0.9)			
				
**Tumor size (cm)**				
≤ 2	215 (33.0)	1.000		**< 0.0001**
2-5	283 (43.5)	1.880	1.255-2.865	
≥ 5	101 (15.5)	1.988	1.323-2.951	
Unknown	52 (8.0)			
				
**Nodal status**				
Negative for node metastasis	327 (50.2)	1.000		**< 0.0001**
Positive for node metastasis	320 (49.2)	1.578	1.307-1.922	
Unknown	4 (0.6)			
				
**Total nodes**	625 (96.0)	0.976	0.956-0.996	**0.022**
Unknown	26 (4.0)			
				
**Nuclear grade**				
Small/uniform nuclei	113 (17.4)	1.000		0.421
Intermediate nuclei	315 (48.4)	1.015	0.624-1.725	
Large nuclei	170 (26.1)	1.272	0.868-1.853	
Unknown	53 (8.1)			
				
**ER status**				
ER negative	289 (44.4)	1.000		0.308
ER positive	326 (50.1)	0.903	0.740-1.097	
Unknown	36 (5.5)			
				
**PR status**				
PR negative	294 (45.2)	1.000		0.433
PR positive	302 (46.4)	0.929	0.769-1.114	
Unknown	55 (8.4)			
				
**HER2 status**				
HER2 negative	495 (76.0)	1.000		**0.020**
HER2 positive	109 (16.7)	1.292	1.041-1.586	
Unknown	47 (7.2)			
				
**MAP-tau expression**				
MAP-tau low expression	376 (57.8)	1.000		**0.018**
MAP-tau high expression	104 (16.0)	0.765	0.598-0.957	
Other	171 (26.3)			

* *P *is given for Cox multivariate analysis, statistically significant *P *values (*P *< 0.05) are in bold, trending *P *values are in italics; CI, confidence interval; ER, estrogen receptor; HR, hazard ratio; HER2, human epidermal growth factor receptor 2; PR, progesterone receptor.

### MAP-tau expression pattern in TAX 307S

**Figure 3:**
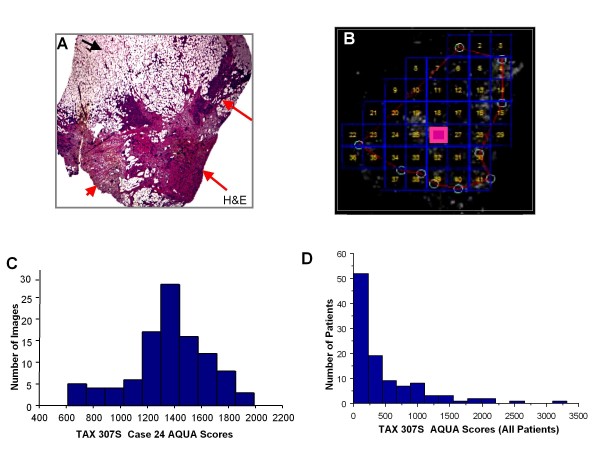
****MAP-tau expression and frequency distribution in TAX 307S whole tissue sections**. (a) **Whole section breast tumor tissue from patient case 24 stained with hematoxylin and eosin showing dark pink staining of tumor cytoplasm compared with blue nuclei staining (red arrows); remaining light pink or non-staining, mesh-like areas comprised of breast adipose tissue (black arrow). Original magnification × 4. **(b) **Whole tissue slide of breast tumor tissue from panel a (case 24) stained with MAP-tau-Cy5 and analyzed using AQUA digital pathology algorithms. A matrix captured 103 quadrants (41 quadrants displayed in panel b and was used to systematically capture each field of view (FOV), creating 103 unique expression scores for case 24. Original magnification × 4. **(c) **Frequency distribution for MAP-tau quadrant scores from case 24 with mean and median AQUA scores of 1357 and 1369, respectively, and a score range of 610 to 1986. FOVs were used to generate a frequency distribution for each case in the TAX 307S cohort (n = 140). The median AQUA score from each case was used in all cohort analyses. **(d) **Distribution of MAP-tau expression in TAX307.

In the TAX 307S metastatic cohort, MAP-tau expression was measured in each WT section using a matrix comprised of FOVs. All FOVs were collected and AQUA scores were generated, but each region was reviewed on a serial H&E slide to confirm that all FOVs represented infiltrating carcinoma. FOVs with normal breast ducts or ductal carcinoma *in situ *were excluded from the analysis. This process is illustrated in Figures 3a and 3b. Similar to the Yale University cohort, MAP-tau expression in TAX 307S remained localized to the cytoplasmic compartment within the epithelial tumor area. A total of 15,816 individual, non-overlapping FOVs were evaluated in the TAX 307S cohort. A frequency distribution summarizing the FOVs was generated for each case in the TAX 307S cohort (Figure 3c). The median score from all FOVs from each case was used to represent that case in the final subset of 108 cases. The distribution of MAP-tau expression in TAX307 for a single patient case is illustrated in Figure 3d. The median level of normal MAP-tau expression as previously applied in the Yale University cohort was used to differentiate high from low expressers, and we observed 32% (35 cases) expressing high MAP-tau compared with 68% (73 cases) showing low expression in TAX 307S (Table 5). This result is consistent with the MAP-tau expression distribution in the Yale University cohort (22% high MAP-tau and 78% low MAP-tau expression; *P *= 0.21 and *P *= 0.43, respectively).

**Table 5:** Correlation between MAP-tau expression and clinicopathologic variables in TAX 307S

	MAP-tau expression		
		
Variable	Low (%) (AQUA score < 462)	High (%) (AQUA score > 462)	P*
**Menopausal status**			
Premenopausal	15 (17.1)	9 (10.2)	0.487
Postmenopausal	45 (51.1)	19 (21.6)	
			
**Tumor size (cm)**			
≤ 2	13 (12.8)	11 (10.8)	0.137
2-5	40 (39.2)	16 (15.6)	
≥ 5	15 (14.7)	7 (6.9)	
			
**Nodal status**			
Negative for node metastasis	23 (24.2)	10 (10.5)	0.922
Positive for node metastasis	41 (43.2)	21 (22.1)	
			
**Tumor grade**			0.140
Well/moderately differentiated	19 (21.1)	12 (13.3)	
Poorly/undifferentiated	45 (50.0)	14 (15.6)	
			
**ER status**			
ER negative	32 (35.6)	8 (8.9)	**0.005**
ER positive	26 (28.9)	24 (26.7)	
			
**PR status**			
PR negative	30 (34.5)	11 (12.6)	0.103
PR positive	26 (29.9)	20 (22.9)	
			
**Prior adjuvant chemotherapy**			
No therapy	30 (27.8)	25 (23.2)	**0.002**
Yes therapy	43 (39.8)	10 (9.3)	
			
**Prior adjuvant hormonal therapy**			
No therapy	47 (43.5)	24 (22.2)	0.666
Yes therapy	26 (24.1)	11 (10.2)	
			
**Prior metastatic hormonal therapy**			
No therapy	56 (51.9)	24 (22.2)	0.371
Yes therapy	17 (15.7)	11 (10.2)	
			
**Response to therapy**			
Complete response (CR)	6 (5.9)	2 (1.9)	0.518
Partial response (PR)	30 (29.7)	18 (17.8)	
Stable disease (SD)	20 (19.8)	9 (8.9)	
Progressive disease (PD)	13 (12.9)	3 (2.9)	
			
**Treatment**			
FAC^1^	39 (36.1)	15 (13.9)	0.303
TAC^2^	34 (31.5)	20 (18.5)	

*P is given for chi-square analysis. Statistically significant *P *values (*P *< 0.05) are in bold.

^1^FAC, 5-fluorouracil-doxorubicin-cyclophosphamide combination

^2^TAC, docetaxel-doxorubicin-cyclophosphamide combination

ER, estrogen receptor; PR, progesterone receptor.

In TAX 307S, as in the Yale cohort above, low MAP-tau expression was significantly more frequent in ER-negative cases (35.6%) compared with ER-positive cases (28.9%; *P *= 0.005). MAP-tau expression was also associated with prior adjuvant chemotherapy (anthracyclines only) with low MAP-tau expression most frequently observed in patients receiving adjuvant chemotherapy (39.8%). MAP-tau expression did not correlate with menopausal status, tumor size, tumor grade, nodal status, PR status, response to therapy, treatment arm, or prior adjuvant or metastatic endocrine therapy.

### MAP-tau prognostic value in TAX 307S

**Figure 4:**
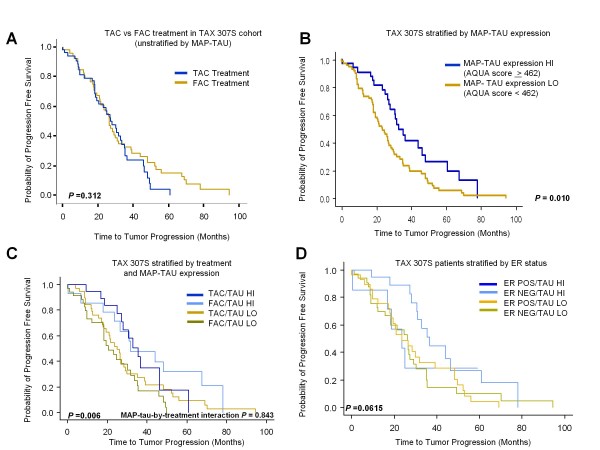
****Progression free survival by treatment arm and MAP-tau status in TAX 307S whole tissue sections**. (a) **Survival analysis for TAC vs FAC-treated patients (not stratified by MAP-tau) in TAX 307S indicated no treatment difference for TTP and confirmed original TAX 307 clinical trial results (n = 484). **(B) **Median progression free survival for patients classified as high MAP-tau expressors (n = 35) was 33.0 v 23.4 months compared with low expressors (n = 73) with mean TTP of 31.2 months (log-rank, *P *= 0.010). **(c) **Median progression-free survival for MAP-tau expression stratified by TAC or FAC treatment showing strong prognostic value for MAP-tau. Progression-free survival of 35.5 and 31.7 months for high MAP-tau expression, regardless of treatment arm (TAC or FAC/Tau high, n = 20, n = 15, respectively) compared with 20.6 and 25.0 months for low MAP-tau expression (TAC or FAC/Tau low, n = 34, n = 39), respectively indicating strong prognostic value for MAP-tau (log-rank, *P *= 0.006). **(d) **Kaplan Meier survival analysis for MAP-tau expression stratified by estrogen receptor (ER) status showing no association between ER status and MAP-tau expression (*P *= 0.0615).

Comparison analysis of TAC vs FAC treatment arms alone (not stratified by MAP-tau) in TAX 307S showed no difference in TTP (*P *= 0.312; Figure 4a) and confirmed original TAX 307 clinical trial results which showed no differences in median TTP (*P *= 0.51) or OS (*P *= 0.93), with a median TTP of 31 versus 29 weeks and median OS of 21 versus 22 weeks for TAC versus FAC, respectively. Improved median five-year DFS was observed for patients in TAX 307 who received TAC versus FAC (69% v 52%; *P *= 0.04) and the inclusion of docetaxel resulted in higher overall response rates for TAC versus FAC (55% vs 44%; *P *= 0.02) [16].

When TAX 307S (TAC plus FAC arms combined) was stratified by MAP-tau expression, we observed prognostic value for MAP-tau with improved median time to PFS for high MAP-tau expressers (n = 35) compared with low expressers (n = 73; 33.0 v 23.4 months) and a mean TTP of 31.2 months (log-rank, *P *= 0.010; Figure 4b), suggesting that this marker maintains prognostic value in metastatic patients.

### MAP-tau predictive value in TAX 307S

Stratification by both treatment arm and MAP-tau expression showed improved TTP for high MAP-tau expression, regardless of treatment, which indicated prognostic but not predictive value for MAP-tau (Figure 4c, log-rank, *P *= 0.006). In addition, no significant interaction between MAP-tau expression and benefit from docetaxel (*P *= 0.843) was observed further confirming the finding of no predictive value for MAP-tau in TAX 307S. High MAP-tau expression was associated with improved PFS (HR: 0.538; 95% CI, 0.333 to 0.871; *P *= 0.011), whereas tumor non-response (stable disease plus progressive disease) was associated with worse PFS (HR: 2.213; 95% CI, 1.404 to 3.488; *P *= 0.0006; Table 6). Multivariate Cox proportional hazards regression analysis showed only high MAP-tau expression was associated with significantly better TTP. For these patients, we observed a 69% reduction of risk of progression (HR = 0.308; 95% CI, 0.130 to 0.728, *P *= 0.007; Table 7).

**Table 6:** Univariate analysis of tumor and clinical risk factors for progression-free survival in TAX 307S

	TAX 307S cohort			
		
Variable	No. of patients (n = 140)	HR	95% CI	P*
**Age**	108 (77.1)	0.977	0.955-1.00	0.060
Other	10 (7.1)			
Unknown	22 (15.7)			
				
**Menopausal status**				
Premenopausal	29 (20.7)	1.000		0.083
Postmenopausal	79 (56.4)	0.627	0.375-1.046	
Other	10 (7.1)			
Unknown	22 (15.7)			
				
**Tumor size (cm)**				
≤ 2	24(17.1)	1.000		0.580
2-5	56 (40.0)	0.728	0.567-1.136	
≥ 5	22 (15.8)	1.134	0.641-2.142	
Other	15 (10.7)			
Unknown	23 (16.4)			
				
**Nodal status**				
Negative for node metastasis	33 (23.6)	1.000		0.119
Positive for node metastasis	62 (44.3)	0.993	0.595-1.249	
Other	23 (16.4)			
Unknown	22 (15.7)			
				
**Tumor grade**				
Well/moderately differentiated	31 (22.1)	1.000		0.163
Poorly/undifferentiated	59 (42.1)	1.412	0.758-1.904	
Other	28 (20.1)			
Unknown	22 (15.7)			
				
**ER status**				
ER negative	40 (28.6)	1.000		0.236
ER positive	50 (35.7)	0.750	0.465-1.208	
Other	28 (20.0)			
Unknown	22 (15.7)			
				
**PR status**				
PR negative	41 (29.3)	1.000		0.847
PR positive	46 (32.9)	0.954	0.592-1.538	
Other	31 (22.1)			
Unknown	22 (15.7)			
				
**Prior adjuvant chemotherapy**				
No therapy	55 (39.3)	1.000		0.983
Yes therapy	53 (37.9)	1.005	0.656-1.538	
Other	10 (7.1)			
Unknown	22 (15.7)			
				
**Prior adjuvant hormonal therapy**				
No therapy	71 (50.7)	1.000		0.512
Yes therapy	37 (26.4)	0.859	0.549-1.349	
Other	10 (7.1)			
Unknown	22 (15.7)			
				
**Prior metastatic hormonal therapy**				
No therapy	80 (57.1)	1.000		0.176
Yes therapy	28 (20.0)	0.711	0.434-1.165	
Other	10 (7.1)			
Unknown	22 (15.7)			
				
**Response to therapy**				
Responder	56 (40.0)	1.000	1.404-3.488	**0.0006**
Non-responder	45 (32.2)	2.213		
Other	17 (12.1)			
Unknown	22 (15.7)			
				
**Treatment**				
FAC^1^	54 (38.6)	1.000		0.315
TAC^2^	54 (38.6)	1.256	0.805-1.960	
Other	10 (7.1)			
Unknown	22 (15.7)			
				
**MAP-tau expression**				
MAP-tau low expression	73 (52.1)	1.000		**0.011**
MAP-tau high expression	35 (25.0)	0.538	0.333-0.871	
Other	10 (7.1)			
Unknown	22 (15.7)			

* *P *is given for Cox multivariate analysis, statistically significant *P *values (*P *< 0.05) are in bold, trending *P *values are in italics;

^1^FAC, 5-fluorouracil-doxorubicin-cyclophosphamide combination

^2^TAC, docetaxel-doxorubicin- cyclophosphamide combination

CI, confidence interval; ER, estrogen receptor; HR, hazard ratio; PR, progesterone receptor.

**Table 7:** Cox proportional hazards multivariate model in TAX 307S

	TAX 307S cohort			
		
Variable	No. of patients (%) (n = 140)	HR	95% CI	P*
**Age**	108 (77.1)	0.972	0.934-1.011	0.153
Other	10 (7.1)			
Unknown	22 (15.7)			
				
**Tumor size (cm)**				
≤ 2	24(17.1)	1.000		0.224
2-5	56 (40.0)	1.116	0.280-4.444	
≥ 5	22 (15.8)	1.326	0.433-4.065	
Other	15 (10.7)			
Unknown	23 (16.4)			
				
**Nodal status**				
Negative for node metastasis	33 (23.6)	1.000		0.207
Positive for node metastasis	62 (44.3)	3.364	0.792-14.297	
Other	23 (16.4)			
Unknown	22 (15.7)			
				
**Tumor grade**				
Well/moderately differentiated	31 (22.1)	1.000		0.773
Poorly/undifferentiated	59 (42.1)	0.897	0.430-1.875	
Other	28 (20.1)			
Unknown	22 (15.7)			
				
**ER status**				
ER negative	40 (28.6)	1.000		0.524
ER positive	50 (35.7)	1.353	0.533-3.424	
Other	28 (20.0)			
Unknown	22 (15.7)			
				
**PR status**				
PR negative	41 (29.3)	1.000		0.751
PR positive	46 (32.9)	1.154	0.475-2.801	
Other	31 (22.1)			
Unknown	22 (15.7)			
				
**Prior adjuvant chemotherapy**				
No therapy	55 (39.3)	1.000		0.604
Yes therapy	53 (37.9)	0.835	0.422-1.650	
Other	10 (7.1)			
Unknown	22 (15.7)			
				
**Prior adjuvant hormonal therapy**				
No therapy	71 (50.7)	1.000		0.810
Yes therapy	37 (26.4)	0.909	0.417-1.980	
Other	10 (7.1)			
Unknown	22 (15.7)			
				
**Prior metastatic hormonal therapy**				
No therapy	80 (57.1)	1.000		0.456
Yes therapy	28 (20.0)	0.714	0.295-1.730	
Other	10 (7.1)			
Unknown	22 (15.7)			
				
**Treatment**				
FAC^1^	54 (38.6)	1.000		0.264
TAC^2^	54 (38.6)	1.517	0.729-3.154	
Other	10 (7.1)			
Unknown	22 (15.7)			
				
**MAP-tau expression**				
MAP-tau low expression	73 (52.1)	1.000		**0.007**
MAP-tau high expression	35 (25.0)	0.308	0.130-0.728	
Other	10 (7.1)			
Unknown	22 (15.7)			

*P is given for Cox multivariate analysis, statistically significant *P *values (*P *< 0.05) are in boldface, trending values are in italics; CI, confidence interval; ER, estrogen receptor; HR, hazard ratio; PR, progesterone receptor.

^1^FAC, 5-fluorouracil-doxorubicin-cyclophosphamide combination.

^2^TAC, docetaxel-doxorubicin-cyclophosphamide combination.

MAP-tau is an ER-regulated gene with inducible expression by both estrogen and tamoxifen, *in vitro *[20]. ER positivity and/or tamoxifen therapy could act by artificially elevating MAP-tau levels and obscuring the relation between MAP-tau expression level and response to taxane therapy. Thus we stratified this cohort by adjuvant endocrine therapy, ER status, and also TAC vs FAC treatment. Stratification by ER status showed a trend, but not significant association between MAP-tau expression and TTP (Figure 4d, log-rank, *P *= 0.0615). Next, we correlated MAP-tau expression levels with response to docetaxel therapy. Response rates as a function of MAP-tau expression did not differ when split by ER status, adjuvant endocrine therapy, or taxane treatment arm (TAC vs FAC; Table 8).

**Table 8:** MAP-tau expression levels and response to docetaxel in TAX 307S

TAX 307S Patient groups	N	Objective response^4 ^(CR + PR) (%)	Stable disease^5 ^(SD) (%)	Progressive disease (PD) (%)	Total	Missing	P*
**ER positive**							0.174
High MAP-tau		15 (33.3)	5 (11.1)	1 (2.2)	21 (46.7)		
Low MAP-tau		10 (22.2)	10 (22.2)	4 (8.9)	24 (53.3)		
Total	50	25 (55.8)	15 (33.3)	5 (11.1)	45 (100.0)	5	
							
**ER negative**							0.721
High MAP-tau		4 (10.5)	4 (10.5)	0 (0.0)	8 (21.0)		
Low MAP-tau		15 (39.5)	13 (34.2)	2 (5.3)	30 (78.9		
Total	40	19 (49.9)	17 (44.8)	2 (5.3)	38 (100.0)	2	
							
**FAC^1 ^and TAC^2^**							0.518
High MAP-tau		20 (19.7)	9 (8.9)	3 (2.9)	32 (31.7)		
Low MAP-tau		36 (35.6	20 (19.8)	13 (12.9)	69 (68.3)		
Total	108	56 (55.4)	29 (28.7)	16 (15.8)	101 (100.0)	7	
							
**TAC^2 ^only**							0.250
High MAP-tau		11 (22.4)	7 (14.3)	1 (2.0)	19 (38.8)		
Low MAP-tau		17 (34.7)	7 (14.3)	6 (12.2)	30 (61.2)		
Total	54	28 (57.1)	14 (28.6)	7 (14.3)	49 (100.0)	5	
							
**TAC^2 ^and ER positive**							0.520
High MAP-tau		9 (45.0)	4 (20.0)	1 (5.0)	14 (70.0)		
Low MAP-tau		2 (10.0)	3 (15.0)	1 (5.0)	6 (30.0)		
Total	20	11 (55.0)	7 (35.0)	2 (10.0)	20 (100.0)	0	
							
**TAC^2^/ER negative/Adj Hormonal -negative^3^**							0.222
High MAP-tau		2 (10.0)	2 (10.0)	0 (0.0)	4 (20.0)		
Low MAP-tau		8 (40.0)	3 (15.0)	5 (25.0)	16 (80.0)		
Total	20	10 (50.0)	5 (25.0)	5 (25.0)	20 (100.0)	0	
							
**FAC^1 ^only**							0.514
High MAP-tau		9 (17.3)	2 (3.9)	2 (3.9)	13 (25.0)		
Low MAP-tau		19 (36.6)	13 (25.0)	7 (13.5)	39 (75.0)		
Total	54	28 (53.9)	15 (28.9)	9 (17.3)	52 (100.0)	2	
							
**FAC^1 ^and ER positive**							0.105
High MAP-tau		6 (24.0)	1 (4.0)	0 (0.0)	7 (28.0)		
Low MAP-tau		8 (32.0)	7 (28.0)	3 (12.0)	18 (72.0)		
Total	28	14 (56.0)	8 (32.0)	3 (12.0)	25 (100.0)	3	
							
**FAC^1 ^and ER negative**							0.919
High MAP-tau		2 (11.1)	1 (5.6)	1 (5.6)	4 (22.2)		
Low MAP-tau		7 (38.9)	5 (27.8)	2 (11.1)	14 (77.8)		
Total	18	9 (50.0)	6 (33.3)	3 (16.7)	18 (100.0)	0	

*P is given for chi-square analysis. Statistically significant *P *values (*P *< 0.05) are in bold.

^1^FAC, 5-fluorouracil-doxorubicin-cyclophosphamide combination

^2^TAC, docetaxel-doxorubicin-cyclophosphamide combination

^3^Adjuvant hormonal therapy-negative patients only

^4^Objective response, complete response (CR) + partial response (PR)

^5^Stable disease defined as minimum of 6 weeks (2 cycles) ER, estrogen receptor; PR, progesterone receptor

## Discussion

Although previous literature shows conflicting results for MAP-tau prognostic and predictive value, in this study of two independent cohorts, we find MAP-tau expression has prognostic value. In both a population-based retrospective cohort, and a randomized clinical trial with taxane as the only variable and uniformly treated metastatic patients, high levels of MAP-tau, a microtubule stabilizing protein, were associated with better outcomes. Increased microtubule stability may be associated with less aggressive tumors. However, the mechanistic reason for this observation is unknown. Furthermore, there is no correlation with nuclear grade in either cohort, suggesting that MAP-tau is not related to the molecular parameters that drive the morphological features contributing to nuclear grade.

Although only one of our cohorts is useful to assess predictive value, we find no evidence for use of this marker as a companion diagnostic test to predict response to taxane therapy. A previous study showed mechanistic evidence for competitive binding between taxanes and MAP-tau [7]. That finding generated the hypothesis that expression levels of MAP-tau would be predictive of the effect of taxanes. However, this study is now the second cohort to fail to show an association between MAP-tau expression levels and response to taxane therapy in the metastatic setting. Although both studies [15] are relatively small, the lack of association in either case raises question regarding the value of measuring MAP-tau in large taxane trials.

Previous studies have shown interactions between taxane therapy and standard breast cancer biomarkers such as ER and HER2 [21-23]. There is a good mechanistic explanation for this finding in the case of ER because MAP-tau is induced by activation of ER by either estrogen or other selective-estrogen receptor modulators (tamoxifen) [20]. When the Yale cohort was stratified by ER status, MAP-tau expression was prognostic only in ER-negative patients. Multivariate analysis indicated that high MAP-tau remained a significant predictor of improved survival for ER-negative patients even after adjusting for tumor size and nodal status. As the ER-negative tumors are presumed to have lost ER function, the presence of MAP-tau may be regulated by other mechanisms. It is also possible that some of the ER-negative cases are really low positives or false negatives. In another study of this cohort, approximately 10% of the ER-negative cases were shown to be ER positive [24]. Furthermore, in an analysis not shown, we find a correlation between ER expression and MAP-tau expression raising the possibility that high MAP-tau is a surrogate for ER. However, the relatively small numbers of patients in these subgroups make it difficult to drawn convincing conclusions. Previous efforts to examine MAP-tau protein expression as a function of ER status have shown no prognostic value [12]. Other studies examining MAP-tau mRNA have also observed no prognostic value when stratified by ER status [11]. However, Andre and colleagues examined mRNA expression in a treated ER-positive cohort and showed low MAP-tau was associated with worse outcome [9,12]. The lack of agreement between these studies may be due to disparities between RNA and protein expression.

## Conclusions

In this study of two independent cohorts, MAP-tau protein expression is shown to be prognostic but has no predictive value for response to docetaxel. The data from this and other studies is based largely on small, single-institution, retrospective studies. However, the lack of a promising result makes it difficult to justify measurement of MAP-tau in the clinical setting or even one of the cooperative group trials for taxane therapy. This is disappointing because a companion diagnostic test for taxane class drugs would be valuable. Future efforts evaluating other MAPs are needed and may be more fruitful.

## Abbreviations

CI: confidence interval; ER: estrogen receptor; FAC: 5-fluorouracil: adriamycin: cyclophosphamide; FOV: field of view; HeCOG: Hellenic Cooperative Oncology Group; HER: human epidermal growth factor receptor; HR: hazard ratio; MAP: microtubule-associated protein; OS: overall serviva; PFS: progression-free survival; PR: progesterone receptor; TAC: docetaxel-doxorubicin-cyclophosphamide; TMA: tissue microarray; TTP: time to progression; WT: whole tissue (slides).

## Competing interests

Drs Rimm and Camp are stockholders in, and consultants to HistoRx, the exclusive licensee of the Yale-held patent on the AQUA technology. Mark Gustavson is an employee of HistoRx and Kimberly Bassi and Franck Appia are employees of Sanofi Aventis.

## Authors' contributions

MTB contributed to the design of the study, collected, analyzed and interpreted the data, and drafted the manuscript. KL and AM assisted with statistical analysis of the data. MG assisted with quantitative data collection. KB and FA provided patient clinicopathologic information. RLC and DLR reviewed patient tumor sections. LH and DLR contributed to the conception and design of the study, data interpretation and drafting of the manuscript. All authors read and approved the final manuscript.

## Supplementary Material

Additional file 1 **Supplemental Table **1**: Yale University cohort characteristics**. Yale University cohort characteristics. Click here for file

Additional file 2 **Supplemental methods**. Description of antibodies, immunofluorescence procedures, and image capture and analysis. Click here for file
