# Supplementary material for: Evaluation of prognostic and predictive value of microtubule associated protein tau in two independent cohorts
Source: Breast Cancer Res. 2011 Nov 2;13(5):R85. doi: 10.1186/bcr2937 (PMC3262195; doi:10.1186/bcr2937)
Supplement: Additional file 1 — Supplemental Table 1: Yale University cohort characteristics. Yale University cohort characteristics. [file bcr2937-S1.PDF]

Supplemental Table 1. Yale University cohort characteristics

Variable	Number (%) (n= 651)
**Age at Diagnosis**	645 (99.1)
Mean age	58.1
Median age	58.0
Range	24 - 88 years old
Unknown/Missing	6 (0.9)
**Menopausal Status**	
Premenopausal	196 (30.1)
Postmenopausal	449 (69.0)
Unknown/Missing	6 (0.9)
**Tumor size**	
<2 cm	215 (33.0)
2-5 cm	283 (43.5)
>5 cm	101 (15.5)
Not specified	52 (8.0)
**Histology**	
Infiltrating ductal carcinoma	520 (79.9)
Infiltrating lobular	14 (2.2)
Carcinoma (nonspecific)	83 (12.7)
Other	34 (5.2)
**Nodal Status**	
Node Positive	320 (49.2)
Node Negative	327 (50.2)
Unknown/Missing	4 (0.6)
**ER**	
Positive	362 (55.6)
Negative	253 (38.9)
Unknown	36 (5.5)
**PR**	
Positive	358 (55.0)
Negative	238 (36.6)
Unknown	55 (8.4)
**HER2**	
Negative	495 (76.0)
Positive	109 (16.7)
Unknown	47 (7.2)
**Treatment**	
Node Positive Patients	Chemotherapy: Adriamycin, Cytoxan, 5-Fluorourac
Node Negative Patients	Local Radiation and Surgical Resection Only
ER Positive Patients	Less than 2% Received Tamoxifen (n=7 patients)
Taxane Treatment	None Received Taxanes
**Censor**	
Censored (20 years)	328 (50.4)
Median follow-up time (years)	21.4
Minimum (months)	4.2
Uncensored (20 years)	276 (42.4)
Unknown	47 (7.2)
**Follow-up time (years)**	
Mean	12.8
Median	8.9
Range	4 months – 41.5 years

Abbreviations: ER, estrogen receptor; PR, progesterone receptor; HER2, human epidermal growth factor receptor 2.

Supplemental Table 2. Clinicopathologic characteristics of TAX 307 versus TAX 307S.

	Cohort		
**Variable**	**TAX 307 (n= 349)***	**TAX 307S (n= 140)**	***P****
**Menopausal Status**			
Premenopausal	61 (17.5)	28 (20.0)	0.674
Postmenopausal	202 (57.9)	83 (59.3)	
Other	86 (24.6)	29 (20.7)	
**Tumor Size (cm)**			
≤ 2	98 (28.1)	31 (22.1)	0.157
2-5	136 (39.0)	66 (47.1)	
≥5	90 (25.8)	30 (21.4)	
	25 (7.2)	13 (9.3)	
**Nodal Status**			
Negative for Node Metastasis	125 (35.8)	42 (30.0)	0.302
Positive for Node Metastasis	187 (53.6)	79 (56.4)	
	37 (10.6)	19 (13.6)	
**Histology**			
Infiltrating Ductal Carcinoma	89 (25.5)	48 (34.3)	0.800
Infiltrating Lobular Carcinoma	125 (35.8)	59 (42.1)	
Other	135 (38.7)	33 (23.6)	
**Tumor Grade**			
Well/Moderately Differentiated	113 (32.4)	44 (31.4)	0.154
Poorly/ Undifferentiated	123 (35.2)	68 (48.6)	
Other	113 ((32.4)	28 (20.0)	
**ER Status**			
ER Negative	81 (23.2)	46 (32.9)	0.179
ER Positive	155 (44.4)	64 (45.7)	
Other	113 (32.4)	30 (21.4)	
**PR Status**			
PR Negative	89 (25.5)	48 (34.3)	0.576
PR Positive	125 (35.8)	59 (42.1)	
Other	135 (38.7)	33 (23.6)	
**Prior Adjuvant Chemotherapy**			
No Therapy	234 (67.0)	71 (50.7)	0.10
Yes Therapy	115 (33.0)	63 (45.0)	
Other	0 (0)	6 (4.3)	
**Prior Adjuvant Hormonotherapy**			
No Therapy	246 (70.5)	90 (64.3)	0.452
Yes Therapy	102 (29.2)	44 31.4)	
Other	1 (0.3)	134-140	
**Prior Metastatic Hormonotherapy**			
No Therapy	255 (73.1)	98 (70.0)	0.974
Yes Therapy	93 (26.6)	36 (25.7)	
Other	1 (0.3)	6 (4.3)	
**Response to Therapy**			
Complete Response (CR)	14 (4.0)	9 (6.4)	0.732
Partial Response (PR)	137 (39.3)	61 (43.6)	
Stable Disease (SD)	104 (29.8)	40 28.6)	
Progressive Disease (PD)	41 (11.7)	17 (12.1)	
Other	53 (15.2)	13 (9.3)	
**Treatment**			
FAC[1]	175 (50.1)	66 (47.1)	0.861
TAC[2]	174 (49.9)	68 (48.6)	
Other	0 (0)	6 (4.3)	

*TAX 307 n=489 but for this analysis the comparison subset (n=140) was removed leaving a total of 349 cases.
**P* is given for chi-square analysis. Statistically significant P values (P < 0.05) are in boldface.
[1]FAC: 5-fluorouracil-doxorubicin-cyclophosphamide combination
[2]TAC: docetaxel-doxorubicin- cyclophosphamide combination
ER, estrogen receptor; PR, progesterone receptor.
