# Supplementary material for: Evaluation of prognostic and predictive value of microtubule associated protein tau in two independent cohorts
Source: Breast Cancer Res. 2011 Nov 2;13(5):R85. doi: 10.1186/bcr2937 (PMC3262195; doi:10.1186/bcr2937)
Supplement: Additional file 2 — Supplemental methods. Description of antibodies, immunofluorescence procedures, and image capture and analysis. [file bcr2937-S2.PDF]

Supplemental Methods

Antibodies and Immunofluorescence:

MAP-tau showed a normal distribution of expression across six sets of Index Arrays with high correlation indicating little batch staining variability (data not shown). To ensure maximum adherence of floated whole tissue sections to glass slides, TAX 307 slides were incubated for 24 hours at 60°C. Oven incubation at 60°C for 20 minutes was used to deparaffinize Yale University TMA slides and Index array slides (with TAX 307 WT slides previously deparaffinized during the 24 hour incubation), followed by two 20 minute incubations in Xylene. After slides were washed twice in 100% ethanol, once in 70% ethanol, and rehydrated with tap water, antigen retrieval by pressure cooking was performed in 6.5 mM sodium citrate buffer (pH 6.0) for 10 minutes. Endogenous peroxidase activity was quenched in methanol with 3% hydrogen peroxide for 30 minutes followed by rinsing in tap water and placement in 1 x trisethanolamine-buffered saline (TBS; pH 8.0). Non-specific binding was reduced using a 30 minute preincubation in 0.3% bovine serum albumin (BSA) in 0.1M tris-buffered saline (TBS, pH=8) with 0.05% Tween (TBS-T). Slides were prepared for 4°C overnight incubation (12 hours) by adding a cocktail of MAP-tau primary antibody (1:750) plus a wide-spectrum rabbit anti-cow cytokeratin antibody (Z0622; DAKO, Carpinteria, CA) diluted 1:100 in BSA/1XTBS-T. Following overnight incubation, slides were washed twice in 1xTBS with 0.05%Tween for 10 minutes and once in 1xTBS. Secondary antibody was then applied for 1 hour at room temperature. Goat anti-rabbit Alexa 488 (Molecular Probes, Eugene OR) was diluted 1:100 in

horseradish peroxidase-conjugated EnVision antimouse secondary antibody (DAKO). Following incubation with secondary antibodies, slides were washed twice (10minutes, then 5minutes) in 1xTBS-T and once (5 minutes) in 1xTBS. Cyanine-5 (Cy5) directly conjugated to tyramide (FP1117, Perkin-Elmer, Boston MA), diluted 1:50 in amplification diluent (Perkin-Elmer) was used as the fluorescent chromagen for target detection and was added to all slides for 10 minutes at room temperature. Two final washes (10minutes, then 5minutes) in 1xTBS-T and one 5 minute wash in 1xTBS were performed. Slides were stained for double-stranded DNA using Prolong Gold mounting medium with anti-fade reagent 4',6-diamidino-2-phenylindole ("DAPI", Molecular Probes, Eugene OR). Serial sections of the specialized Breast Cancer Cell Line control TMA were stained alongside both cohorts to confirm assay reproducibility. Normal breast epithelium in the Yale University cohort TMAs and the TAX 307 WS slides served as internal positive controls while omission of the primary antibody served as the negative control for each immunostaining event.

Image Capture and Analysis:

Protein concentration within specified subcellular compartments can be measured on a continuous scale using Automated Quantitative Analysis (AQUA). In brief, a series of high resolution monochromatic images were captured using the PM-2000™ microscope (HistoRx, New Haven, CT). Fluorescent chromagens (DAPI, Cy2, Cy5) were used to demarcate subcellular compartments within each histospot or whole section image. Areas of tumor epithelia were differentiated

from stromal elements by creating an epithelial tumor mask using cytokeratin-Alexa 488. Binary gating of positively stained cytokeratin pixels, in which pixels are assigned *on* or *off*, results in the accurate representation of tumor area within the histospot or whole section. DAPI staining of cell nuclei was used to generate the nuclear compartment. Subtracting the generated nuclear compartment from the masked cell area created a non-nuclear compartment, which included both the cytoplasm and membrane. To visualize the target, MAP-tau-Cy5 was measured in the tumor mask. RESA and PLACE algorithms, previously described[27], were used to address issues of histospot or tissue section thickness and overlapping compartments. To generate an AQUA score, each pixel was recorded on a scale of 0 (black) to 255 (white) and pixel intensity was defined as: [target pixel intensity] x [compartment pixel intensity/255]. Using the target and compartment pixel intensities, an equation was generated where: [sum of all target pixel intensities] / [sum of all compartment pixel intensities/255] equals the AQUA score. This equation can be summarized as the average signal intensity per unit of compartment area and is expressed on a scale of 0-33,000. Note that the target and compartment pixel intensities are raw integers and this is reflected in the unitless AQUA score.
